# Sorafenib in the management of metastatic renal cell carcinoma

**DOI:** 10.3747/co.v16i0.430

**Published:** 2009-05

**Authors:** C. Guevremont, C. Jeldres, P. Perrotte, P.I. Karakiewicz

**Keywords:** Sorafenib, metastatic renal cell carcinoma, targeted therapy

## Abstract

**Purpose:**

Sorafenib represents one of the two standards of care for patients with metastatic renal cell carcinoma (mrcc). In the present review, we provide information regarding the use of sorafenib in first and second lines. We also describe results for dose escalation strategies. Finally, we provide data addressing the efficacy of sorafenib in patients with mrcc of non-clear-cell histology.

**Recent Findings:**

Sorafenib is a valid first-line agent. Sorafenib response rates and toxicity are not affected by patient age or site of metastasis. The sequence of first-line sorafenib followed by second-line sunitinib resulted in a longer duration of response than did the opposite sequence. Sorafenib efficacy in first-line therapy can be potentiated by co-administration of low-dose interferon. Moreover, in first-line therapy, impressive response rates were recorded when the dose of sorafenib was escalated beyond the standard 400 mg twice daily. Similarly impressive response rates were observed with dose escalation in second-line therapy. It is notable that dose escalation after failure of standard sorafenib dose also prolongs progression-free survival. Finally, the efficacy of sorafenib is not limited to clear-cell histology, but also applies to chromophobe and papillary mrcc variants.

**Summary:**

Sorafenib is a highly effective and well-tolerated agent for first- and second-line patients with clear-cell, chromophobe, or papillary mrcc variants.

## 1. INTRODUCTION

Until the advent of targeted therapies, interferon alfa and interleukin-2 represented the standards of care for patients with metastatic renal cell carcinoma (mrcc). Since 2006, six randomized controlled trials have demonstrated the efficacy of five novel targeted therapies in primarily clear-cell mrcc patients 1–6.

First, in previously untreated patients (*n* = 750), Motzer *et al.* showed that, as compared with interferon, sunitinib improves median progression-free survival (pfs) to 11 months from 5 months [hazard ratio (hr): 0.42; *p* < 0.001] 1. Then, in patients previously exposed to interferon (*n* = 903), Escudier *et al.* showed that, as compared with placebo, sorafenib improves median pfs to 5.5 months from 2.8 months (hr: 0.44; *p* < 0.001) 2. Subsequently, in previously untreated patients (*n* = 626), Hudes *et al.* showed that, as compared with interferon, temsirolimus improves median overall survival (os) to 10.9 months from 7.3 months (hr: 0.73; *p* = 0.008) 3. Thereafter, in previously untreated patients (*n* = 649), Escudier *et al.* showed that, as compared with interferon alone, bevacizumab combined with interferon improves median pfs to 10.2 months from 5.4 months (hr: 0.63; *p* = 0.0001) 4. The efficacy of bevacizumab was corroborated by Rini *et al.* (*n* = 732), who observed an increase in pfs to 8.5 months from 5.2 months (hr: 0.71; *p* < 0.0001) 5. Most recently, in patients who experienced failure of one or more previous targeted therapies (*n* = 410), Motzer *et al.* showed that, as compared with placebo, everolimus improves median pfs to 4.0 months from 1.9 months (hr: 0.31; *p* < 0.0001) 6. Taken together, these findings illustrate the efficacy of targeted therapies in first, second, and subsequent treatment lines.

In the present manuscript, we focus on sorafenib, and we review data supporting its use in the first, second, and subsequent treatment lines. Moreover, we show data that support the use of sorafenib in patients with non-clear-cell histology. Finally, we review data demonstrating increased efficacy when the dose of sorafenib is escalated beyond the usual 400 mg twice daily.

## 2. CURRENT GUIDELINES IN THE TREATMENT OF MRCC

The National Comprehensive Cancer Network (nccn) 7, the European Association of Urology 8, and the European Organisation for Research and Treatment of Cancer (eortc) 9 guidelines all suggest the use of sunitinib in the first line for favourable- and intermediate-prognosis mrcc. In patients with poor-prognosis mrcc, temsirolimus is recommended in the first line.

Based on the findings of the Escudier *et al.* randomized controlled trial of sorafenib versus placebo after cytokine failure, treatment with sorafenib is recommended in patients with clear-cell histology after failure of cytokine therapy. The standard recommendation for sorafenib use is therefore in second-line therapy 2. However, according to the eortc and nccn recommendations, select patients may be candidates for sorafenib in first-line treatment 7,9.

## 3. EVIDENCE OF SORAFENIB EFFICACY AND FAVOURABLE TOXICITY

### 3.1 Phase I Sorafenib Studies

Sorafenib is a multikinase inhibitor, specifically targeting tumour cells and tumour vessels. In its original design, the molecule was developed as a specific inhibitor of the Raf-1 protein kinase. In subsequent studies, sorafenib was found to have selective activity against B-Raf, vascular endothelial growth factor receptors 2 and 3, platelet-derived growth factor receptor β, Flt-3 (fms-like tyrosine kinase 3), and stem-cell growth factor C-Kit 2,10.

The safety and toxicity of sorafenib were investigated in four phase i studies (*n* = 173). Doses ranged from 50 mg to 800 mg once or twice daily 11–14. The maximal tolerated dose was 400 mg given continuously twice daily. Dose-limiting toxicities—grade 3 or higher according to the National Cancer Institute (nci) Common Toxicity Criteria (ctc) version 2.0 15—consisted of diarrhea (2 of 6 at 800 mg twice daily), fatigue (1 of 6 at 800 mg twice daily), and skin toxicity (4 of 14 at 600 mg twice daily). Based on the efficacy-to-toxicity ratio, the 400-mg twice-daily dosage, which was associated with a manageable toxicity profile, was recommended as the target dose for future trials.

### 3.2 Phase II Sorafenib Trials

The efficacy of sorafenib was demonstrated in a phase ii placebo-controlled randomized discontinuation trial of sorafenib in 202 patients with mrcc. Sorafenib-exposed patients had a significantly prolonged pfs (24 weeks vs. 6 weeks, *p* = 0.0087). Moreover, during the run-in phase, an investigator-assessed twice-daily dimensional tumour assessment in 193 evaluable patients (from among all patients exposed to sorafenib) showed stable disease (sd) in 34% and partial responses (prs) in 36% 16.

### 3.3 Phase III Sorafenib Studies

A phase iii sorafenib trial was conducted in 903 patients previously treated with immunotherapy; these patients were randomized either to placebo or to sorafenib 400 mg twice daily. The most common adverse events of any grade [according to the nci Common Terminology Criteria for Adverse Events (ctcae) version 3.0 17] were diarrhea (43%), rash or desquamation (40%), fatigue (37%), hand–foot skin reaction (30%), alopecia (27%), and nausea (23%). The most frequent grades 3 and 4 toxicities included hand–foot skin reaction (6%), fatigue (5%), dyspnea (4%), and hypertension (4%). Grade 3 or 4 anemia occurred in 3% of patients, and grade 3 or 4 lymphopenia occurred in 13% of patients. Neutropenia was not reported. Cardiac ischemia or infarction occurred in 3% of patients 2.

Relative to placebo, sorafenib improved median pfs to 5.5 months from 2.8 months (hr: 0.44; *p* < 0.001) 2. The efficacy of sorafenib with regard to quality-of-life outcomes and toxicity profile were all unaffected by patient age 18–21. Similarly, sorafenib efficacy was unrelated to the site of metastasis 22.

#### 3.3.1 Pitfalls in the Interpretation of Cancer Control Features in the Phase III Sorafenib Trial

In accordance with the ethical considerations that apply to placebo-controlled trials, placebo patients who demonstrate disease progression are allowed to cross over to the active treatment arm after disease progression has been documented. Such a measure reduces the recorded efficacy of active therapy and dilutes its effect. This phenomenon affected os and pfs data within the sorafenib trial 19.

For example, before placebo-arm patients crossed over to the sorafenib arm, the initial analysis demonstrated a reduction in overall mortality that resulted in a hr of 0.72 (*p* = 0.02) 2. In the analysis that was performed after crossover of the placebo patients into the sorafenib arm, the hr decreased to 0.77 (*p* = 0.02) 2. Finally, at completion of the study, when even more placebo crossovers had occurred, the hr further decreased to 0.88, with a loss of statistical significance as evidenced by *p* = 0.146 19.

Taken together, these observations illustrate the confounding effect of crossover that cannot be controlled with statistical tools. Crossovers confound the magnitude and significance of cancer control measures so profoundly that os and pfs measures performed after crossover cannot be validly interpreted. It is questionable whether a non-censored survival analyses should be performed after crossover from a placebo arm has occurred. Consequently, study designs with censoring at crossover might represent a better alternative. In the analysis of sequential therapies, os and pfs should be invariably interpreted before any crossover.

## 4. FIRST-LINE USE OF SORAFENIB

First-line use of sorafenib was examined in only one randomized study, a phase ii trial conducted by Escudier *et al.* (*n* = 189) that was reported in 2009 23. Its design addressed pfs and toxicity of sorafenib relative to interferon. The median pfs was similar in both trial arms, 5.7 months with sorafenib as compared with 5.6 months with interferon [hr (interferon/sorafenib): 0.88; *p* = 0.5]. The disease control rate [complete response (cr) + pr + sd ≥ 6 weeks: 79.4%] achieved with sorafenib was higher than that achieved with interferon (64.1%, *p* = 0.006). Health-related quality of life was better with sorafenib than with interferon.

Despite the randomized nature of this trial, several caveats limit its value. First, the interferon data were inconsistent with other median pfs values for interferon. For example, in the percy Quattro study by Negrier *et al.* 24, a median pfs of 3.4 months was recorded for patients exposed to interferon. Second, the Escudier *et al.* study 23 was underpowered because of its premature closure. The pfs findings are therefore difficult to interpret.

Two phase ii studies examined the combination of sorafenib with interferon in first-line therapy 25,26. Ryan *et al.* 25 (*n* = 62) co-administered 400 mg twice-daily sorafenib with interferon 10×10^6^ IU. This combination resulted in a median pfs of 7.0 months, but toxicity was dose-limiting. Tannir *et al.* 26 (*n* = 72) explored the efficacy of first-line sorafenib 400 mg twice daily with low-dose interferon (0.5×10^6^ IU twice daily) and recorded a pfs duration of 7.6 months. The toxicity was marginally more elevated in the combined sorafenib–interferon arm, with a 61.5% dose reduction rate as compared with a 51.2% rate for sorafenib alone. These data illustrate a promising increase in the efficacy of first-line sorafenib when co-administered with interferon (pfs: 5.7 months vs. 7.0–7.4 months) 25,26. Moreover, only a marginal increase in toxicity should be expected when interferon is co-administered in a twice-daily low dose.

## 5. SORAFENIB DOSE ESCALATION

Based on phase i and ii data, the standard dose of sorafenib was defined at 400 mg twice daily 2. This dosing is usually very well tolerated and allows for safe dose escalation in most patients 2. Two studies assessed the effect of dose escalation on response rates in first- or second-line therapy 27,28.

In 2007, Amato *et al.* 27 studied 44 patients previously exposed to a maximum of one systemic therapy and recorded a median pfs of 8.4 months. Overall, 91% of the patients tolerated dose escalation up to 1200 mg or 1600 mg daily. Impressive antitumour activity was observed, as shown by 8 crs and 14 prs for a total cr+pr rate of 52%. Because most of the adverse events were grade 1 or 2, toxicity was highly acceptable.

In 2008, Amato *et al.* 28 studied a second cohort of 23 patients who had previously been exposed to a maximum of one cytokine therapy. Of those patients, 87% had no exposure to any previous therapy. Overall, 22 of 23 patients tolerated escalation to 1200 mg daily, and 14 of 14 patients tolerated 1600 mg daily. An equally impressive pfs of 7.7 months was recorded. However, the combined cr+pr rate of 32% was lower than that seen in the initial study (52%). Again, most of the adverse events were grade 1 or 2.

Recently, Escudier *et al.* 23 (*n* = 189) studied the effect of dose escalation in second-line therapy after failure of sorafenib 400 mg twice-daily dosing. At progression, the dose was escalated to 600 mg twice daily, and a 41.9% tumour response rate was recorded. An additional 39.5% of patients showed sd. The median pfs was 3.6 months despite initial progression on sorafenib. The combined median pfs of 5.7 months after first-line standard sorafenib dosing, and the subsequent median pfs of 3.6 months after sorafenib dose escalation, resulted in an impressive 9.3 months of combined median pfs, with an overall response rate of 84%.

In a multicentre prospective trial, Shepard *et al.* 29 also studied the effect of sorafenib dose escalation after failure of regular sorafenib 400 mg twice-daily dosing. Patients (*n* = 42) had previously experienced progression after sunitinib or bevacizumab-based therapy. The sorafenib dose was escalated to 600 mg or 800 mg twice daily in 9 patients; these patients subsequently required dose reductions. Overall, this study demonstrated a tumour burden reduction rate of 31% and a sd rate of 53%. Median pfs was 3.7 months, which is consistent with the findings reported by Escudier *et al.* (pfs: 3.6 months). Side effects were manageable, and the toxicity profile of dose-escalated sorafenib in this study was similar to that seen in other trials 2,23.

Taken together, the dose escalation data show that in the first line, sorafenib delivered at doses that exceed the 400 mg twice-daily standard dose may result in a cr rate of up to 20% 23. Such a high proportion of crs has never previously been reported. However, those promising results need to be corroborated in large-scale trials. In the second line, sorafenib dose escalations are equally promising and demonstrate that the median pfs may be increased by at least 3 months if either sorafenib or another targeted therapy was administered before dose escalation 23,27–29.

## 6. SORAFENIB SEQUENTIAL THERAPY

Data from a randomized controlled trial confirm the efficacy of sequential sorafenib therapy after cytokine failure 2. In contemporary patients, cytokines are almost never used as monotherapy, particularly in the light of the percy Quattro data that showed the efficacy of interferon to be equivalent to that of medroxyprogesterone 24. However, data on the use of sorafenib after failure of other targeted therapies (which could justify contemporary guidelines for second-line use) are scarce.

Knox *et al.* 20 were the first to report on efficacy of sorafenib sequential therapy from the North American Advanced Renal Cell Carcinoma Sorafenib Access Program (arccs). Within that program, 1255 patients received sorafenib as second-line or subsequent therapy. Interferon alfa, interleukin-2, bevacizumab, sunitinib, and thalidomide were previously administered to 646 (51%), 522 (42%), 290 (23%), 24 (2%), and 142 (11%) of those patients respectively. For patients taking sorafenib in the second or a subsequent line, the rates of pr and sd were 3% and 81% respectively 20. Those rates compared very favourably with the 4% pr and 79% sd rates seen in first-line sorafenib patients within the same program 20.

Sablin *et al.* 30 were the first to report on differences in response rates according to the sequence of agents used. In that study, sequential therapy with first-line sorafenib and subsequent sunitinib (*n* = 68) was associated with better results (median pfs: 26 weeks) than was first-line sunitinib with subsequent sorafenib (*n* = 22; median pfs: 22 weeks) 30.

Recently, Dudek *et al.* 31 (*n* = 49) reported on the use of sequential therapy with sorafenib and sunitinib. Their data showed a combined median pfs of 78 weeks for patients initially treated with sorafenib followed by sunitinib as compared with 37 weeks for patients initially treated with sunitinib followed by sorafenib (risk ratio: 3.0; *p* = 0.016). The authors explained the observed difference in survival as being the result of a stronger resistance to targeted therapy after initial sunitinib exposure as compared with after initial sorafenib exposure.

The findings of Sablin *et al.* 30 and Dudek *et al.* 31 are consistent with the report by Motzer *et al.* 6 on the efficacy of everolimus after failure of previous targeted therapy. Motzer *et al.* demonstrated that everolimus results in a pfs of 5.9 months after sorafenib failure as compared with 3.4 months after sunitinib failure 6. In consequence, superior pfs results can be expected if sorafenib instead of sunitinib is used as the first targeted therapy.

Most recently, Tamaskar *et al.* 32 reported a combined pr+sd rate of 71% in 14 patients exposed to sorafenib after various targeted therapy failures.

Taken together, these findings support the use of first-line sorafenib as recommended by the nccn and eortc guidelines 7,9. However, the sequence of targeted therapies remains to be corroborated in larger-scale trials.

## 7. USE OF SORAFENIB IN NON-CLEAR-CELL RENAL CARCINOMA

In the randomized phase iii sorafenib placebo-controlled trial, 100% of patients harboured clear-cell histology 2. Since the publication of that report, a number of investigators have showed that excellent response rates may also be expected in patients with the papillary or chromophobe histologic variants 2,20,32,33.

Knox *et al.* 20 represent the first investigator group to address the efficacy of sorafenib in non-clear-cell histology. The arccs expanded access program included 118 individuals with papillary and 18 with chromophobe histology. The combined pr+sd rate for papillary mrcc was 80% as compared with 95% for the chromophobe variant 20.

Choueiri *et al.* 33 showed a 68% sd rate in 28 individuals with papillary mrcc. As with the arccs data 30, the pr+sd rate in chromophobe non-clear-cell rcc patients (*n* = 5) was high at 100%.

Predominantly based on Choueiri *et al.* 33 and the arccs 20 data, the efficacy of sorafenib in papillary and chromophobe mrcc variants may be expected to parallel the efficacy reported for clear-cell histology. The extent to which this effect may be modified by treatment line (first vs. second vs. subsequent), or by co-administration of biologic response modifiers such as interferon, remains to be seen.

## 8. CONCLUSIONS

In the present manuscript, we have demonstrated that sorafenib represents a valid first-line agent 23,24,27. The sequence of first-line sorafenib followed by second-line sunitinib results in longer response duration than does the opposite sequence 20. Moreover, sorafenib efficacy in first-line therapy can be potentiated by co-administration of low-dose interferon 25. Also, in first-line therapy, impressive response rates have been recorded when the dose of sorafenib was escalated beyond the standard 400 mg twice daily 27,28. Similarly impressive responses were observed with dose escalation in second-line therapy 27,28. Notably, dose escalation after failure of a standard sorafenib dose also prolonged pfs 23. Finally, the efficacy of sorafenib is not limited to clear-cell histology, but also applies to the chromophobe and papillary mrcc variants 20,33. Last but not least, a comparison of toxicities between sorafenib, sunitinib, and temsirolimus demonstrated that sorafenib is associated with the lowest toxicity rates, which further validates the use of sorafenib as the initial targeted agent 34.
